# Metabolomics-Assisted Breeding in Oil Palm: Potential and Current Perspectives

**DOI:** 10.3390/ijms25189833

**Published:** 2024-09-11

**Authors:** Rizki Anjal P. Nugroho, Ismail Zaag, Emmanuelle Lamade, Rudy Lukman, Jean-Pierre Caliman, Guillaume Tcherkez

**Affiliations:** 1 Institut de Recherche en Horticulture et Semences, Université d’Angers, 49070 Beaucouzé, France; rizkianjalpuji.nugroho@etud.univ-angers.fr (R.A.P.N.); ismael.zaag@inrae.fr (I.Z.); 2 Sinar Mas Agro Resources and Technology Research Institute (SMARTRI), Jalan Teuku Umar 19, Pekanbaru 28112, Riau, Indonesia; rudy.lukman@sinarmas-agri.com (R.L.); j.p.caliman@sinarmas-agri.com (J.-P.C.); 3 Centre de Coopération Internationale en Recherche Agronomique pour le Développement, UMR ABSYS, 34398 Montpellier, France; emmanuelle.lamade@cirad.fr; 4 Systèmes de Pérennes, University of Montpellier, Centre de Coopération Internationale en Recherche Agronomique pour le Développement, 34398 Montpellier, France; 5 Research School of Biology, Australian National University, Canberra, ACT 2601, Australia

**Keywords:** oil palm, crosses, progenies, metabolic pathways, metabolomics

## Abstract

Oil palm is presently the most important oil-producing crop worldwide in terms of oil production and consumption. However, oil palm cultivation faces important challenges such as adverse climatic conditions, expensive fertilization requirements, and fungal pathogens, including *Ganoderma*. Intense efforts in oil palm breeding are devoted to improving both oil production yield and resistance to environmental cues. Metabolomics can be of interest because it provides many quantitative traits and metabolic signatures that can be selected for to optimize oil palm performance. Here, we briefly review how metabolomics can help oil palm breeding, and to do so, we give examples of recent metabolomics analyses and provide a roadmap to use metabolomics-assisted breeding.

## 1. Introduction

Oil palm (*Elaeis guineensis* Jacq.) is the primary oil crop worldwide, with more than 80 Mt of oil produced each year (www.fao.org/faostat, URL accessed on 8 September 2024). Due to the demand of the food industry, intense efforts are devoted to increasing oil palm performance, not only in terms of crude oil production but also in terms of nutrient use efficiency and environmental value. There is, therefore, strong interest in new methods of breeding to obtain and release elite oil palm lines commercially. Amongst breeding methods, the use of metabolomics was suggested several years ago because it may provide access to quantitative traits (metabolites as molecular markers) implementable for genetic association studies or selection of oil palm germplasms [1,2,3]. Metabolomics refers to the integrative analysis of metabolic compounds so as to identify marker metabolites associated with diseases, nutrient use efficiency, genetic origin, etc. In doing so, metabolomics informs us of metabolic pathways that are expressed under specific conditions and, thus, are useful to understand physiological responses and assess metabolic differences between plant lines. In oil palm, there has been a number of successful uses of metabolomics to identify biomarkers of, e.g., infection by, or resistance to *Ganoderma* [4,5,6], drought stress [7], or mineral nutrition [8,9,10]. Also, oil palm has an enormous potential for metabolic markers because of the broad diversity of metabolites (metabolic pathways) expressed in this species. In fact, this species does not only produce diverse compounds of lipid metabolism (involved in oil production) but also nitrogenous (alkaloids, catecholamines) and non-nitrogenous (benzoic acid derivatives, flavonoids) secondary metabolites in leaves [11,12,13,14]. In this Perspectives paper, we provide an update on the potential of metabolomics utilization to assist oil palm breeding, not only to differentiate oil palm families and thus select genetic lines of interest but also to screen palms with high-performance traits such as nutrient use efficiency. We first recall the basics of oil palm breeding, then summarize the use of metabolomics in this species, and, finally, assess how metabolomics could be used as a tool (with pros and cons) for modern breeding programs.

## 2. Brief History of Oil Palm Breeding

**Figure 1:**
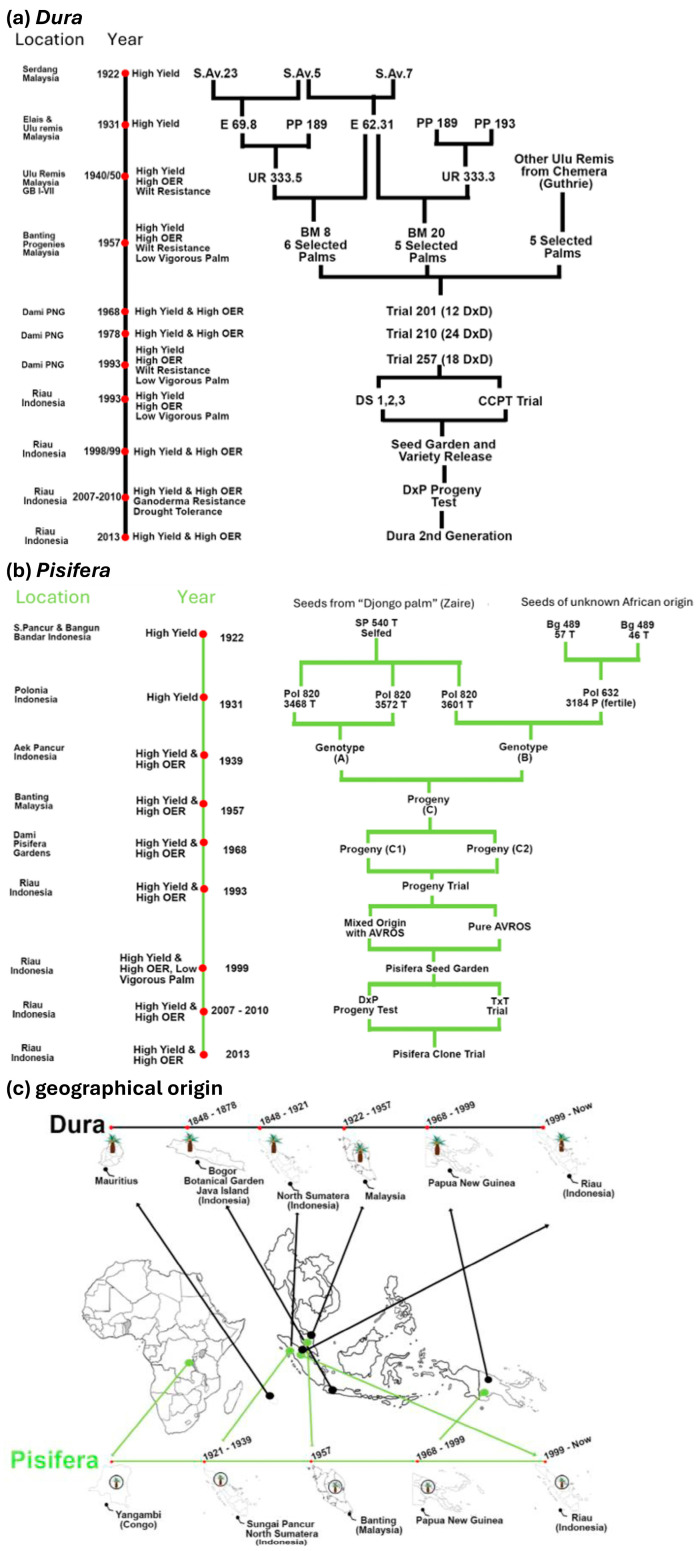
Breeding history of oil palm in Indonesia, which has generated *Dura* and *Pisifera* palms cultivated nowadays. Simplified crossing scheme and genealogy are shown in panels (**a**) and (**b**) for *Dura* and *Pisifera* palms, respectively. The geographical origins are displayed on a map in panel (**c**). Note that the African origin of modern oil palms can be traced back for *Pisifera*. Crossing history is simplified and thus shows main events, important dates, and breeders. OER, oil extraction rate.

Oil palm families are currently differentiated based on fruit types: *Dura*, *Pisifera*, and *Tenera* [15]. In most crossings, *Dura* palms are used as a female parent while *Pisifera* are used as a male parent, generating *Tenera* hybrids, which represent the vast majority of palm trees in plantations for oil production [16]. A majority of *Dura* palm breeding involved Southeast Asia and started from palms introduced in the Bogor Botanical Gardens (Indonesia) by the Dutch government in 1864 (Figure 1). Thus, we focus here on the origin of oil palms used in Indonesia (mostly in Sumatra) as an example (for a description of *Deli* and *La Mé* origins from Angola and Democratic Republic of Congo and associated breeding history, see [17,18,19,20]). After the initial introduction of *Dura* palms, breeding yielded several lines known as, e.g., *Marihat Baris*, *Rispa*, *Dolok Sinumbah*, and, then, *Deli*, produced after several breeding cycles. Similarly, several *Pisifera* varieties bred in Southeast Asia started from Djongo palms from Yangambi (Congo). *Yangambi* palms were then planted in Sungai Pancur and yielded the family referred to as SP540. *Avros* populations now used routinely by several breeding teams have been produced via breeding cycles from SP540 [21].

The breeding method implemented for oil palm has changed significantly with time and depends on oil palm type (Dura, Pisifera and Tenera) [2,16,22]. The first breeding method exploited mass selection, and it is reviewed in [15]. Briefly, the best palms, i.e., palms with traits of interest such as fresh fruit bunches production (FFB) or oil extraction rate (OER), were selected from broad, random populations. This method might have been associated with potential bias in that diverse environmental conditions influenced selection because some palms could have been located in better environments and, thus, performed better (i.e., yielded more fruit bunches) than others due to environment and not genotype effects [23,24]. The next method was modified reciprocal recurrent selection (MRRS), which has been used to obtain “elite”, very high-yielding palm lines. MRRS evaluates both parent and progeny performance in controlled agronomic trials to identify elite palms (within *Dura* or *Pisifera* populations) or elite hybrid *Tenera* palms in progenies and select the best parental lines. MRRS may also combine with family individual palm selection (FIPS) for elite Tenera palms. Using iterative crossing, the MRRS/FIPS method can be used to generate elite palms.

## 3. Overview of Current Breeding Strategies

Currently, conventional breeding methods implemented in plantations are inherited from MRRS/FIPS and thus are tedious due to high cost, high time consumption, and genotyping. The high cost of oil palm breeding is the consequence not only of high land surface area required for trials but also of the intensive manpower resources required for field maintenance and data recording. Also, conventional oil palm breeding with successive generations implies several growing cycles, representing about 20 years from characterization until elite *Tenera* palms can be released [23,25]. From a breeding or genetic point of view, oil palm collections used nowadays are made of many germplasms still partly linked to wild populations and co-ancestral genetic composition. On the one hand, the genetic structure of oil palm shows heterogeneity between palms of the same populations/family and between populations/families [17,20,26] with relatively elevated heterozygosity. That is, the usual breeding programs do not have near-isogenic lines to work with, and furthermore, *Tenera* lines of interest are hybrids from *Dura* and *Pisifera*. Also, many wild alleles are still present in selected genotypes. On the other hand, family populations are relatively similar genetically, that is, with many common haplotypes [20], because originally, only a few palms were used as starting material (Figure 1), narrowing the genetic diversity. As such, conventional oil palm breeding is of “low intensity”, i.e., selects for various genetic assemblages and, therefore, does not yield pure homozygous genotypes. Recently, oil palm breeding has taken advantage of biotechnological and robust statistical approaches to increase selection intensity with low cost and time consumption and improved accuracy. Best linear unbiased prediction (BLUP) is one of the statistical tools that are being used in oil palm breeding for evaluation of unbalanced mating design and precise breeding value [27,28]. Genomic selection prior to crossing is also implemented as a new method for efficient oil palm breeding. It has been utilized, via simultaneous genomic and phenotypic data, to select the best *Tenera* and parental lines; see, e.g., [24,29,30,31]. There is still a high demand for breeding methods in oil palm to obtain the best *Tenera* hybrids and parental populations with minimal cost and time and maximal precision and selection intensity.

## 4. The Potential of Metabolomics in Oil Palm

**Figure 2:**
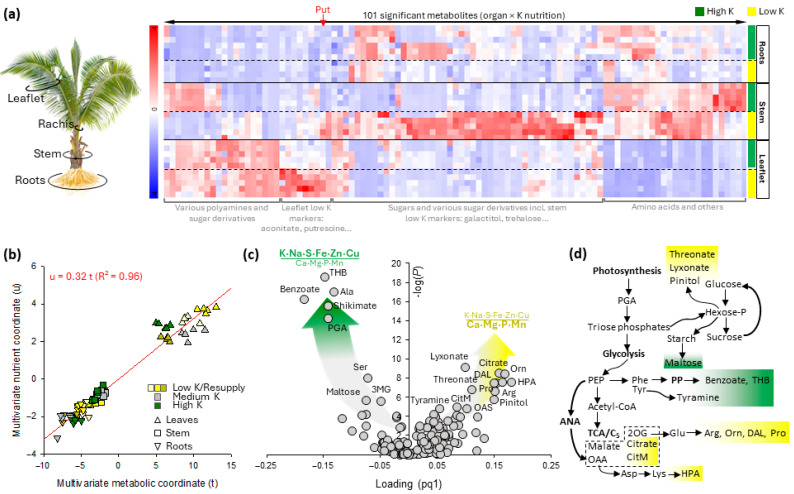
Evidence for a metabolomic response to nutrient conditions in oil palm, exemplified by the effect of K availability in leaflets, stem, and roots of one-year-old saplings. (**a**) Heatmap view of metabolites associated with a significant tissue × K availability effect, showing organ-specific nutrient responses. (**b**) Bi-block OPLS multivariate analysis showing the correlation between the multivariate nutrient variable (denoted as *u*) and multivariate metabolic variable (denoted as *t*), regardless of conditions across all organs. (**c**) Volcano plot associated with panel (**b**), showing the *p*-value of the regression (F-test) against the OPLS loading to make apparent best metabolic drivers of the nutrient balance. (**d**) Simplified metabolic scheme replacing best driver metabolites in metabolic pathways. 3MG, 3-methylglutarate; ANA, anaplerotic fixation; CitM, citramalate; DAL, δ-aminolevulinate; OAA, oxaloacetate; PEP, phosphoenolpyruvate; PGA, 3-phosphoglycerate; PP, phenylpropanoid pathway; THB, trihydroxybenzoate; HPA, hydroxypicolinate. The typical potassium-responsive metabolite putrescine is shown in panel (**a**) with a red arrow. This figure has been redrawn from [8,10].

**Figure 3:**
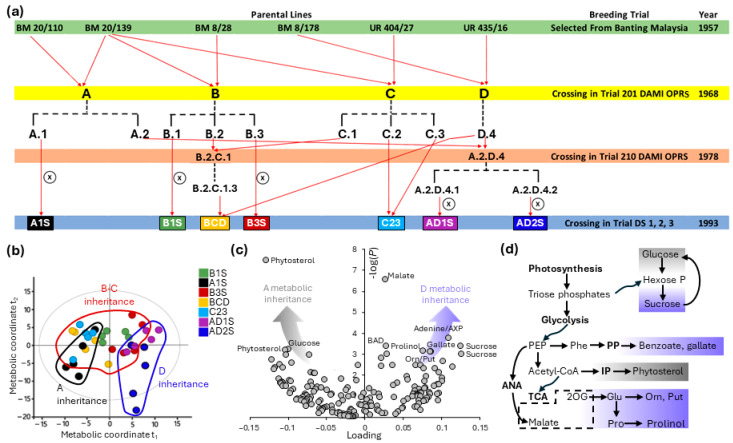
Evidence for line-dependent metabolome in oil palm, exemplified by leaflet ^1^H-NMR analysis in oil palm of different crossing histories cultivated in the same plantation in Indonesia. (**a**) Crossing origin of selected palm families. (**b**) OPLS multivariate analysis showing the discrimination of palms of A and D ancestries, while palms from other ancestors (B, C) appear as intermediates along axis 1 (main discrimination axis). (**c**) Volcano plot showing the *p*-value (ANOVA) against the OPLS loading to make apparent line-specific metabolites. (**d**) Discriminating metabolites replaced in major metabolic pathways. 2OG, 2-oxoglutarate; AXP, adenosine nucleotide; BAD, benzoic acid derivative (e.g., conjugated to glucose). The circled cross (⊗) stands for intra-family crossing (as opposed to hybridization between families). Redrawn from [11,43].

Metabolomics has been used extensively in oil palm biochemistry or in other research fields related to oil palm; in effect, a search using the terms “oil palm” and “metabolomics” together retrieved about 4000 articles in Google Scholar. Amongst them, many papers cite rather than use metabolomics. Many metabolomics analyses carried out in oil palm so far deal with the effect of the fungus *Ganoderma* [4,5,6] or abiotic stress such as drought [7], waterlogging [10], phosphorus deficiency [32], or potassium deficiency [10,11,33,34]. Also, metabolomics methods focused on lipid analysis (lipidomics) have been used recently to monitor fatty acid synthesis and associated pathways during fruit development [35,36]. Metabolomics take advantage of nuclear magnetic resonance (NMR), gas chromatography–mass spectrometry (GC–MS), or liquid chromatography–mass spectrometry (LC–MS). Untargeted metabolomics based on MS usually generate a high number of features (each feature being characterized by the retention time and the mass-to-charge ratio, *m*/*z*), leading to a high number of variables that can be inspected via, e.g., multivariate analysis, to assess correlation with the trait of interest (nutrient content, resistance to infection, etc.). In the figures provided below, we use orthogonal partial least squares (OPLS) as an example of multivariate analysis, using nutrient content (Figure 2) or oil palm family (Figure 3) as a trait with which to correlate metabolic features.

Metabolomics-assisted breeding in oil palm can be envisaged only if the oil palm metabolome does effectively and quantitatively link to traits of interest that can be selected for. Here, the term “link” is used to refer to either prediction (i.e., the metabolic profile is a good predictor of production such as OER or FFB) or responsiveness (i.e., the metabolic profile reflects tolerance to stress such as nutrient deficiency, water restriction, pathogen attack, or nutrient use efficiency). In addition, metabolomics analyses must be carried out relatively easily on samples that can be accessed on a routine basis.

In the case of oil palm, it is highly desirable to use leaflets, rather than fruit, trunk, or root samples, because they are already used routinely to describe tree nutrient status via the elemental content in K, N, or P of vegetative tissue sampled with very standardized protocols (e.g., leaflet sampled at point B of a given leaf rank) [37,38,39]. Also, using leaflets, as opposed—or in addition—to fruit metabolic signatures, would be useful because it would not be necessary to wait several years, until the production phase, to assess oil palm lines; that is, analyses could be conducted at the vegetative stage. Additionally, leaflets effectively show a metabolome response to environmental conditions (while other organs also show change in metabolome), as will become apparent in the example provided in Figure 2.

To our knowledge, despite published suggestions to exploit metabolomics for breeding [1], there is limited use of metabolomics signatures of OER or FFB, while genomic association analysis of oil production parameters has been undertaken, e.g., in [40,41]. In a recent analysis, three *Tenera* (*Deli* × *La Mé*) progenies (non-referenced and of unknown origin) of contrasted OER were compared [42]. Although this study is obviously associated with the confounding effect of genetics (i.e., progeny/crossing effect), it suggests that fruit sucrose content (along with other sugars and other compounds such as tryptamine and tyramine) correlates with OER (the correlation coefficient between sucrose and oil yield was about 0.9, with a very low *p*-value, and sucrose alone explained about 3% of oil yield variance in the multivariate analysis).

To date, the most extensive metabolomics analysis of oil palm was carried out on its response to potassium availability. In trees grown in the field, an indirect (i.e., K-driven) relationship between FFB and metabolome was found [33,34]. Although these studies do not reflect direct links between metabolic signatures and production traits, they provide a quantitative assessment of the response to K availability, which is a critical parameter of oil production. Under controlled nursery conditions, oil palm nutrient (potassium) status, which can be represented by the K·Na·S·Fe·Zn/Ca·Mg·P·Mn balance, may be monitored via metabolic signatures (summarized in Figure 2) [8,10,11]. Interestingly, clear biomarkers of nutrient imbalance could be identified in several organs (such as ornithine, putrescine, tyramine, and citramalate), and similarly, good nutrient balance markers have been found (such as benzoate, trihydroxybenzoate, and shikimate) (Figure 2). Importantly, machine-learning statistical algorithms have suggested that such biomarkers are more accurate than leaflet K elemental content itself [8], demonstrating that the ability of palm trees to incorporate potassium into metabolism (i.e., effective K use efficiency) is directly accessible via metabolites. Also, a detailed study investigating biomass allocation and nutrient composition in several oil palm organs pointed out that there was no simple quantitative elemental trait that reflects the physiological tree K balance or FFB [44]. These pieces of information are crucial because they show the importance and usefulness of marker metabolites and, thus, of specific metabolic pathways (illustrated in Figure 2d) to access actual K utilization by oil palm. This means that, potentially, metabolic pathways or candidate marker metabolites could be selected for to obtain optimal K-efficient oil palm lines.

In the past few years, metabolomics analyses have been conducted to identify metabolites that are markers of infection by *Ganoderma* (*Ganoderma orbiforme* (Fr.) Ryvarden, also referred to as *G. boninense*, a major pathogen of oil palm in both Asia and Africa). For example, leaf rachis has been shown to be enriched in 2-oxoglutaramate and phosphocholine in infected palms [6], while leaflets have been shown to contain more chelidonic acid, along with other secondary compounds, in both partially resistant and susceptible palm lines [5]. Disease severity has been found to correlate with several metabolites of core pathways (including amino acids aspartate and asparagine) and several intermediates of arginine and urea metabolism (arginine, creatinine, allantoin), suggesting that infection by *Ganoderma* requires a specific reorchestration of N metabolism [45]. Interestingly, amongst metabolic differences between monokaryotic (less pathogenic) and dikaryotic (more pathogenic) mycelia of *Ganoderma*, arginine (as well as proline and phenylalanine) and S-containing metabolites have been found [46]. The potential role of secondary metabolites, including derivatives of fatty acids and phenylpropanoids, has also been discussed [4,12]. Thus, there is some potential for marker metabolites to allow early detection of infection by *Ganoderma* and, perhaps, to locate metabolic pathways that could be targeted via breeding methods or gene editing to minimize susceptibility or infection development. Using data from pre-nursery palm seedlings, a QTL analysis found genome regions associated with resistance to *Ganoderma*, suggesting, accordingly, that genetic marker-assisted selection might be possible for breeding programs [47]. That said, there is presently limited knowledge on marker metabolites associated with *resistance* rather than the difference between healthy and infected palms. The comparison of *Dura*, *Pisifera*, and *Tenera* palms and crosses with contrasted susceptibility to *Ganoderma* has shown a link between resistance and some metabolites, in particular, glycerol and ascorbic acid, that were found to be candidate biomarkers discriminating moderately resistant *Dura × Pisifera* crosses from susceptible *Dura* and *Pisifera* [48]. Also, a comparison of oil palm progenies of different geographical origins (Congo × Cameroon and Nigeria × La Réunion/Mauritius) which exhibit contrasted resistance to *Ganoderma* has suggested that some lipids, including sitosterols and tocopherols, along with nitrogenous compounds (pyrimidines, pyridines), could be candidate biomarkers of resistance [49]. Nevertheless, the use of distant populations leads to strong differences in both genetic composition and plant properties, representing a confounding factor in statistical analyses. Therefore, future studies will have to identify metabolic biomarkers of resistance more precisely, using comparable oil palm populations.

## 5. Practical Imperatives for Metabolomics-Assisted Breeding of Oil Palm

In practice, metabolomics-assisted breeding can use metabolism in two ways: (i) metabolomics signatures obtained via LC–MS, GC–MS, or NMR analyses can be used as environmental response or disease resistance markers to select oil palm lines of interest (regardless of genetic markers that can be determined separately), or (ii) metabolomics signatures associated with specific genetic markers can be used as surrogates of genomic selection to monitor crossings and line selection. At first glance, (i) and (ii) may appear similar. However, utilization (i) is more adapted when no strong genetic markers have been identified, i.e., when the palm traits of interest, including metabolic composition, are highly multigenic. Nevertheless, implementing metabolomics for breeding of oil palm is associated with several practical imperatives, as follows:

- First, there should be good candidate metabolome-based descriptors of traits of interest. The case of K use efficiency is illustrated above in Section 4 and suggests that building a quantitative multivariate variable that can be used to monitor traits is possible (Figure 2b,c). In the case of nutrient utilization, this represents a remarkable opportunity, since it provides access to a complex trait. In the case of potassium nutrition, assessing potassium utilization by trees is a long and resource-consuming process, generally involving agronomic trials at some stages to define a line- and -environment-dependent critical %K in leaflets [37], differential decomposition of growth response curves [50], or plantation K balance [51]. In other words, there could be a universal metabolic signature that can be used to anticipate physiological palm K sufficiency without the need to conduct long and costly agronomic trials required by other methods. However, to date, uncertainty remains as to whether reliable metabolic descriptors will be accessible for traits other than K use efficiency. Additionally, it is worth noting that a candidate metabolic marker is reliable when the factor of interest (drought, nutrient availability, etc.) varies while other parameters are controlled to avoid bias due to the involvement of common metabolites in several responses to environmental cues (i.e., the influence of confounding factors).
- Second, there must be natural variations amongst oil palm families and genotypes in metabolic pathways, making possible associations between genetic markers and metabolites. That is, under the assumption that the traits of interest are determined by metabolic properties, differences in metabolic content can be used to select oil palm lines and perform breeding.

Also, the diversity in metabolomes is reflective of differentially expressed metabolic pathways between oil palm families or progenies, and this can be exploited to maximize metabolic pathways of interest. An important case to consider is the expression of the metabolic pathway associated with lipid synthesis in developing fruits, which must be maximal (as opposed to fibers and sugars) in selected lines to improve OER (which is between 19 and 28% across the world’s oil palm plantations).

Transcriptomics analysis of *Dura*, *Pisifera*, and *Tenera* hybrids has suggested that a role is played by WRINKLED1 [52], a transcription factor that has been shown to be involved in the balance between lipid, protein, and sugar in developing seeds in *Arabidopsis* [53]. Co-expression analysis conducted in a backcross population from *E. guineensis* × *oleifera* hybrids has also shown the tight link between fatty acid synthesis and sugar utilization and starch synthesis in fruits [54]. This indicates that the metabolic partitioning of compounds imported from phloem sap in fruits is crucial for the lipid content at maturity. In the above-cited study [42], sucrose and other sugars in fruits also correlated with OER when three *Tenera* progenies were compared. Overall, it is thus probable that specific *leaflet* metabolomics signatures correlate to OER, such as specific sugar species reflective of sugar export to fruits via the phloem (e.g., raffinose or galactinol [11]), which can be regulated in oil palm leaflets despite the predominance of sucrose as the source used by developing fruits or embryos [55,56,57].

Preliminary metabolomics data obtained to date using diverse oil palm families cultivated under identical conditions in plantations effectively indicate that there are natural variations in metabolome (Figure 3). In this example, the comparison of oil palm families with contrasted breeding histories (dating back to crossings made in 1957) demonstrates the genetic dependence (i.e., heritability) of leaflet metabolomes. In particular, a specific metabolic signature of D inheritance is visible, with increased natural contents in polyamines, sucrose, malate, and benzoate (Figure 3c). It is remarkable that there is some overlapping between nutrient-sensitive pathways (Figure 2d) and naturally differentially expressed pathways (Figure 3d). This shows the potential of expressed metabolic pathways as a criterion to select oil palms that have an intrinsic ability to respond efficiently to nutrient availability. In terms of carbon utilization, it is worth noting that the balance between glucose and sucrose relates to fruit production and allocation to bunches [58,59,60], and therefore, the natural diversity between families observed in the present example may reflect a contrasted ability to partition sugars to developing fruits.

**Figure 4:**
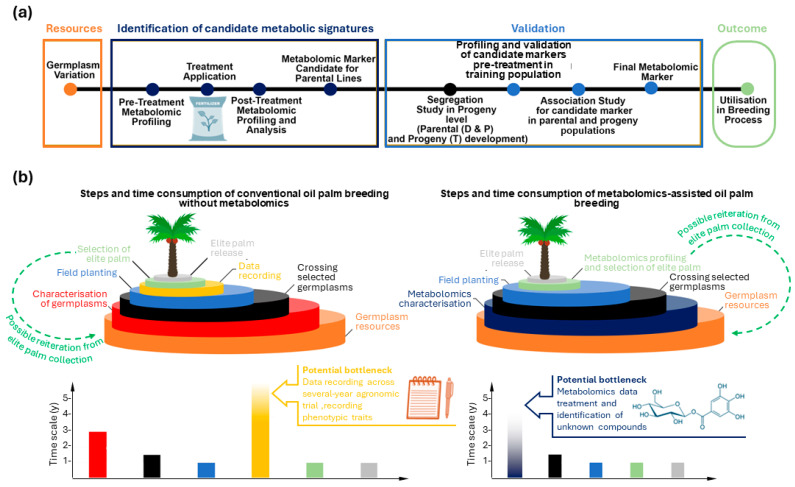
Simplified scheme illustrating metabolomics-assisted breeding of oil palm and how it compares to conventional breeding. (**a**) Steps involved in metabolomics-assisted breeding when the response to an environmental factor has to be taken into account as a selected trait (here, illustrated with fertilization response); therefore, this workflow differentiates pre-treatment (pre-fertilization) and post-treatment (post-fertilization) steps and metabolic profiles. (**b**) Comparison of conventional breeding steps (left) and anticipated steps of metabolomics-assisted breeding (right), with estimated time durations (bottom). The possibility of reselection from a first generation of elite palms is shown in dashed green. Major potential bottlenecks (rate-limiting steps) in the process are shown with large arrows (see main text for further explanations). In the case of conventional breeding, the step associated with data recording and agronomic trials can take up to 9 years.

- Third, metabolomics analysis and interpretation themselves must be relatively fast. In fact, should metabolomics be a slow step, it would compensate for the time gained by bypassing long agronomic trials and data recording. That is, in the breeding process (illustrated step by step in Figure 4), uncertainty remains as to whether metabolomics themselves might represent a bottleneck. This is an important question because metabolomics-assisted breeding requires metabolomics analysis, adding steps associated with both identification of candidate metabolome signatures (dark-blue steps, Figure 4a) and validation (light-blue steps, Figure 4a). It is unlikely that metabolomics analyses per se would be highly time-consuming. In particular, rapid methods like ^1^H-NMR can be used, allowing acquisition of well-resolved spectra within 10 min and identification and quantification of many metabolites, including sugars, amino acids, catecholamines, and polyamines, in oil palm [11,45]. Also, automated exact mass spectrometry by GC–MS can perform derivatization prior to injection, facilitating sample processing and acquisition [61]. LC–MS analyses can also be performed relatively rapidly (about 25 min per sample) including on crude extracts. For a recent example, see [46]. However, data treatment and extraction can be relatively long (Figure 4b, dark-blue bar) for LC–MS datasets, due to spectral cleaning (elimination of adducts, noise *m*/*z* signals, etc.) and compound annotations. Recent methods have been developed for routine LC–MS data trimming, such as MS-CleanR [62], that facilitate the simplification and integration of relevant metabolomics features. However, annotation is still challenging.

There are recent efforts in combining compound databases such as Pharmakon [63], but automatic annotation of secondary metabolites is often prone to errors (up to 35% misidentification). In the recent, publicly available database for palm trees, ArecaceaeMDB [64], 1274 metabolites (including 300 lipid species) are listed for oil palm, made of 204 known molecules (identified metabolites), 1050 putative metabolites, and 20 unknowns. Overall, oil palm compounds not only include primary metabolites (sugars, amino acids, etc.) but also many secondary metabolites like benzoic acid derivatives, phenylpropanoids, and alkaloids. Future studies are warranted to improve automatic annotation plug-ins with already known and/or genome-predicted metabolic pathways in oil palm.

## 6. Conclusions and Perspectives

Taken as a whole, there is good potential in metabolomics-assisted breeding. Currently, genomic selection approaches are used to assist oil palm breeding. As mentioned in the introduction, to date, the use of metabolomics as a selection tool in oil palm breeding is not the subject of published research nor is it carried out in practice. That said, there are examples of its use in other monocots, in particular, in maize (*Zea mays* L.), where metabolic signatures have been used for GWAS analysis and/or breeding [65,66,67,68]. For example, metabolomics could help gene mining in maize breeding associated with resistance to salt stress [68]. Presently, in the case of oil palm, several important traits that are highly dependent on metabolism could be selected for breeding, such as resistance to *Ganoderma* and high nutrient use efficiency. Also, in the future, other key aspects will have to be addressed, such as tolerance to environmental conditions driven by climate change, occasional drought, or bushfires and changes in cultural practices like intercropping [69,70].

It is also worth noting that breeding and selection can be assisted by new technologies such as genome editing, allowing the modification of metabolic properties that are important for agronomic traits. In particular, the CRIPR/Cas9 technology represents a good opportunity to generate palm lines where metabolic pathways can be reorchestrated to improve production (for a review, see [71]). Recently, oil palm lines with increased content in oleic acid have been generated via CRIPR/Cas9 targeted to genes encoding for enzymes of lipid metabolism, fatty acid desaturase 2, and palmitoyl-acyl carrier protein thioesterase [72]. However, applying genome editing technology to metabolism-based oil palm traits is currently limited by our knowledge of metabolic regulations in oil palm and the identification of solid candidate metabolic markers. Therefore, future studies are warranted to fill this gap in knowledge via metabolomics and physiological analyses to identify key metabolic pathways and intermediates that are critical for oil palm agronomic traits such as nutrient use efficiency or resistance to *Ganoderma*.
